# Prevalence of Prolonged Grief Disorder and Related Clinical Factors During the COVID-19 Pandemic in Turkey

**DOI:** 10.1192/j.eurpsy.2024.215

**Published:** 2024-08-27

**Authors:** R. Tekdemir, A. Kandeger, Ö. Tan, M. Aydın, K. Altinbas

**Affiliations:** ^1^Psychiatry, Selcuk University Faculty of Medicine, Mazhar Osman Mood Clinic; ^2^Psychiatry, Selcuk University Faculty of Medicine, Konya; ^3^Freelance, Consulting doctor, Istanbul, Türkiye

## Abstract

**Introduction:**

Prolonged grief disorder has recently been added to the Diagnostic and Statistical Manual of Mental Disorders 5, Text Revision. To understand the health burden and then allocate economic and professional resources, it is necessary to provide epidemiological data for this new disorder. More information on the characteristics of people suffering from PGD is also beneficial to better identify individuals at risk.

**Objectives:**

This study, therefore, aimed to estimate the prevalence of the PGD criteria in a special period such as the Covid-19 pandemic and in a representative population-based sample, evaluate the sociodemographic, and loss-related correlates of PGD caseness and explore possible predictors.

**Methods:**

The study included 126 people (97 females/29 males) who lost a relative for any reason during the Covid-19 pandemic period (March 2019-January 2022) in Turkey.We used self-reported data from articipants who all completed questions on socio-demographic and loss-related characteristics plus Hospital Anxiety and Depression Scale (HADS), Prolonged Grief Disorder Scale (PG-13), Multidimensional Scale of Perceived Social Support (MSPSS), Adult Separation Anxiety Questionnaire (ASA-27).

**Results:**

Median age was 34 years, range (18-63); 12 participants were diagnosed with PGD (9.5%). No difference was detected between deaths due to COVID-19 and its complications and deaths due to other causes in terms of PGD diagnosis and PGD symptom severity. When we divide the participants into two groups according to PGD diagnosis (PGD and nonPGD):The average age of the PGD group was higher (Z=-2.068; p=0,31) and they had more additional medical conditions (χ²=7.21; p=0,007). Thoughts of guilt were more common in the PGD group (χ²=7.92; p=0,005). Additionally, HADS-total, HADS -depression, HADS -anxiety and ASA-27 were higher in the PGD group (respectively: Z=-4.047; P=0,00, Z=-4.209; P=0,00, Z=-3.437; P=0,001, Z=-1.975; P=0,048). PGD occurred most frequently after first-degree losses (χ²=13.67; p=0,00) and was inversely proportional to the age of the loss (Z=-1.979; P=0,04). In the nonPGD group, the rate of believing in any religion (χ²=5.807; p=0,016). and the level of fulfilling the requirements of the religion were higher ( χ²=10.584; p=0,05). In the linear regression analysis examining the predictors associated with the severity of prolonged grief; the deceased person was a first-degree relative (t= 6.23; p<0,001) and younger in age (t=-3.71; p<0,001), the presence of guilt (t= 3.28; p=0,001), and increased separation anxiety (t= 4.13; p<0,001) and depression scores (t= 4.29; p<0,001) were significant boost of prolonged grief severity.

**Conclusions:**

Although higher PGD rates were expected in deaths due to Covid-19 compared to deaths due to other causes, we did not detect any significant difference in this study. However, this study identified some possible predictors associated with PGD.

**Disclosure of Interest:**

None Declared

